# The Oral Microbiome and Systemic Health: Bridging the Gap Between Dentistry and Medicine

**DOI:** 10.7759/cureus.78918

**Published:** 2025-02-12

**Authors:** Subash Chandra Nayak, P. Bhagya Latha, Bharath Kandanattu, Unni Pympallil, Ankit Kumar, Harish Kumar Banga

**Affiliations:** 1 Department of Conservative Dentistry and Endodontics, Hi-Tech Dental College and Hospital, Bhubaneshwar, IND; 2 Department of Zoology, SIR C R Reddy College, Eluru, IND; 3 Pediatric and Preventive Dentistry, Institute of Dental Studies and Technologies, Modinagar, Ghaziabad, IND; 4 Department of Prosthodontics, Mahe Institute of Dental Sciences & Hospital, Mahe, IND; 5 Dentistry, Mithila Minority Dental College and Hospital, Darbhanga, IND; 6 Fashion and Lifestyle Accessory Design, National Institute of Fashion Technology, Kangra, IND

**Keywords:** bacterial oral microbiota, dental medicine discipline, oral health, periodontitis, pregnancy

## Abstract

The oral microbiome, consisting of a mixture of bacteria, fungi, and viruses, is an important contributor to oral and systemic health. Microbial balance disruptions are associated with oral pathologies like dental caries and periodontitis as well as systemic diseases such as cardiovascular diseases, adverse pregnancy outcomes, and respiratory diseases. This review explores the mechanistic pathways linking oral dysbiosis to systemic inflammation, endothelial dysfunction, and immune modulation. The roles of key microbial species in health and disease are analyzed, with an emphasis on how hematogenous dissemination leads to systemic pathologies through inflammatory signaling. Also, advances in high throughput sequencing are discussed, as well as microbial diversity and its implications for diagnostics and therapeutics. The review highlights the potential of oral microbiota-targeted interventions to mitigate systemic diseases through dentistry and medicine integration, by throwing light on interdisciplinary strategies. Future work should focus on the evaluation of the mechanisms by which the oral microbiome plays a role in systemic diseases through the integration of multi-omics approaches such as metagenomics, transcriptomics, and metabolomics. Furthermore, clinical trials need to be designed in a way to evaluate the efficacy of microbiome-targeted therapies in the prevention of cardiovascular diseases, adverse pregnancy outcomes, and autoimmune disorders.

## Introduction and background

The oral microbiome comprises all the genetic material of all the microorganisms that live in the oral cavity, bacteria, viruses, fungi, and protozoa, while the oral microbiota represents the microbial community present in the oral cavity, including both identified and unidentified microorganisms in various oral niches, i.e. teeth, tongue, gingiva, and saliva [1]. Research over the past decade has steadily shown strong associations between periodontal diseases and systemic conditions such as cardiovascular diseases, pregnancy complications, rheumatoid arthritis, or Alzheimer's disease [2-4]. Overall, these findings emphasize the need to adopt a multidisciplinary method that brings together oral drugs and systemic healthcare.

Many well-documented pathways have explained the impact of oral microbes on systemic health. The most strongly supported mechanism explains hematogenous dissemination as the means of delivery of periodontal pathogens (e.g. Porphyromonas gingivalis, Aggregatibacter actinomycetemcomitans) into the bloodstream where the organisms initiate a systemic inflammation capable of incapacitating endothelial function and damaging organs [5]. Second is molecular mimicry, whereby one of the ways the bacterium manages to escape attack is by being sufficiently like existing tissues on the inside so that the immune system attacks the right tissues instead. Other pathways to be examined are those between gut microbiome interactions, i.e., how the oral microbiota impacts the composition of the gut microbiome over immune responses and ultimately in metabolic and inflammatory disorders. Moreover, sharing of oral microbiota can impact digestion and nutrition and consequently bring on systemic metabolic change and inflammation, especially in the gut and cardiovascular system [6].

**Figure 1:**
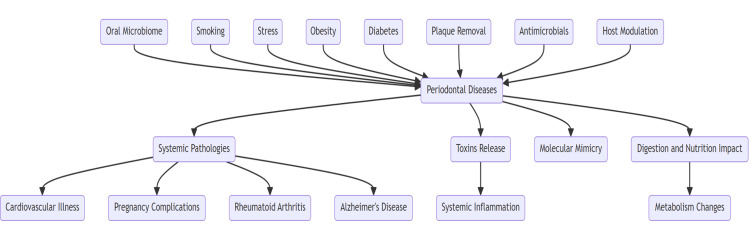
The impact of oral microbiome on systemic health Image credit: Subash Chandra Nayak

Periodontal disease is correlated with other chronic conditions and there are both modifiable and nonmodifiable risk factors. The periodontal tissue flora is adversely affected by smoking and local inflammation is exacerbated, while stress, obesity, and diabetes predispose to dysbiosis and increased inflammatory responses in the periodontium, which further amplify systemic pathology [7]. Furthermore, since periodontal therapy consists of plaque removal, antibiotic treatment, and host modulation, it will aid in reducing systemic inflammatory mediations and overall organ burden [8]. Nevertheless, to solidify the available body of evidence, further research pursued in collaboration between dentistry, medicine, and microbiology is needed, as illustrated in Figure 1.

However, these current insights alone already provide official recommendations for better collaboration of dentists with physicians for better prevention, early detection, and early treatment of oral-systemic connections. Periodontal care should be discussed with these at-risk patients and medical practitioners must perform oral cavity examinations, followed by creating sound referral systems [9]. The studies also suggest that periodontists require more information on the medical management of such patients. Finally, bringing an end to this historical split between the mouth and the rest of the body will create new possibilities for the betterment of public health in general.

## Review

Composition of the oral microbiome

Roles of Bacteria, Fungi, and Viruses in Oral Health

The oral microbiome is a highly diverse community of microorganisms, bacteria, fungi, viruses, and archaea living in different oral cavity niches [10]. Several bacterial species have been identified from the oral cavity using high throughput sequencing such as metagenomic shotgun sequencing and 16S rRNA sequence 700+ [11]. Oral microbiome composition is site-specific with different microbial communities present in different sites such as teeth, gingival sulcus, tongue dorsum, inner cheeks, hard and soft palate, and tonsillar crypts [12]. It is noteworthy that molar surfaces are the sites of the most diverse microbial populations because of their anatomical structure and the biofilm retaining capacity, while the inner cheek and hard palate harbor a less diverse microbial population [13].

Supragingival plaque (above the gum line) is more conducive for the growth of Streptococcus spp. and Actinomyces spp. On the other hand, because of the low oxygen levels in subgingival plaque (under the gum line), anaerobic bacteria, such as Porphyromonas, Prevotella, and Fusobacteria, dominate [14]. Anaerobic bacteria are found in greater numbers in the posterior region of the tongue and dorsal surface, as well as in the tonsillar crypts compared with other oral sites [15]. Of note, the oral microbiome composition is dynamic and changes throughout the developmental stages from birth [16].

Clinical Relevance and Microbial Functional Roles

These microbial species play a big role in health and the progression of disease according to recent research. Streptococcus mitis and Veillonella parvula are commensal bacteria that facilitate oral homeostasis by maintaining pH levels, and synthesis of antibiotics or antagonism against pathogenic colonization [14]. However, like the periodontopathogens Porphyromonas gingivalis and Tannerella forsythia, these bacteria influence dysbiosis, immune evasion, and inflammatory tissue destruction to lead to periodontal disease progression [15]. Under immunocompromised conditions, fungal members of the microbiome can change from a commensal to a pathogenic state, leading to oral candidiasis and denture stomatitis [16]. Furthermore, viral species such as Epstein-Barr virus (EBV), herpes simplex virus (HSV), and human papillomavirus (HPV) have been reported for oral mucosal lesions, gingival inflammation, and higher incidence of oral squamous cell carcinoma in rabbits [17].

Advancements in Microbiome-Targeted Therapeutics

Presently, advances in metagenomic and multi-omics analyses have also led to a better understanding of microbial interactions along with their impact on the modulation of disease and treatment of strategies. To restore bacteriologic balance and decrease oral infections, studies in microbe-restricted intervention using antimicrobial peptides, probiotic therapy, and modulating the host are ongoing [18]. The microbiome studies must be followed up in the future by longitudinal microbiome sequencing and by clinical trial testing of microbiome-based treatment and its effects on systemic health.

Key Microbial Species in Oral Cavity

When dental prosthetics or enamel are newly exposed to the mouth, for example, the first bacteria to adhere to the surfaces are planktonic streptococci [17]. For example, Streptococcus oralis, Streptococcus mitis, and Streptococcus sanguinis start by binding to the acquired pellicle, a glycoprotein-rich layer created from saliva. They modify their local environment to allow the adhesion and growth of other microbial species, which eventually lead to the formation of oral biofilms [18].

Actinomyces spp. are the initial colonizers in supragingival plaque formation and adhere to the acquired pellicle with the help of early colonizers such as Streptococcus sanguinis and Streptococcus oralis [4]. Other facultative anaerobes including Neisseria spp., Haemophilus spp., and Rothia spp. also coaggregate with streptococci and are involved in the formation of a complex biofilm on the tooth surface above the gum line [19].

On the other hand, the subgingival plaque is predominantly composed of gram-negative anaerobic rods which are the major etiological agents in periodontal disease. Periodontitis is strongly associated with Porphyromonas gingivalis, Prevotella intermedia, Tannerella forsythia, and Aggregatibacter actinomycetemcomitans which can evade host immune responses, degrade connective tissues, and stimulate chronic inflammation through immune activation by lipopolysaccharide [20].

The oral pathologies are also due to fungal colonization, and Candida albicans represent about 80% of the fungal infections in the oral cavity, causing oral thrush and denture stomatitis [20]. Oral diseases that have been associated with viral infections, including Epstein Barr virus (EBV), herpes simplex virus (HSV), human papillomavirus virus (HPV), and hepatitis viruses, including EBV to oral ulcers and leukoplakia and raised susceptibility to oral squamous cell carcinoma [20].

The Uses of Bacteria, Fungi, and Viruses in the Dental Part of Oral Health

The majority of oral bacteria live as part of highly structured microbial biofilms that are important to oral and systemic health. The roles of these commensal microbes include regulation of pH balance, facilitation of processes involving nutrient metabolism, modulation of the immune responses, and prevention of colonization of pathogenic species via the principles of microbial interference and bacterial antagonism. For example, by competing for adhesion sites, hydrogen peroxide, and bacteriocins, they contribute to the inhibition of the proliferation of pathogenic bacteria in the oral cavity, gastrointestinal tract, and upper respiratory system [11].

During commensal oral streptococcal growth, conditions are created that prevent the establishment of Aggregatibacter actinomycetemcomitans, Prevotella intermedia, and other periodontopathogens via hydrogen peroxide production. This action is antimicrobial, being mediated by oxidative stress-induced bacterial DNA damage and disruption of anaerobic bacterial metabolism resulting in the decreased ability of bacteria to form biofilm and contribute to periodontal disease [12]. Subgingival anaerobes produce antimicrobial substances and promote the prevention of dysbiosis in subgingival biofilm formation [6]. Indiscriminate use of broad-spectrum antibiotics results in an indiscriminate kill of pathogenic and beneficial bacteria in the oral microbiome thus upsetting the microbial equilibrium. These commensal bacteria, Streptococcus salivarius and Veillonella parvula among them, are depleted causing also a reduction of microbial competition, and of the protective mechanisms mediated by bacterial antagonism and immune modulation. This causes opportunistic pathogens, including Candida albicans and Clostridioides difficile to overgrow and predispose to oral infections like oral candidiasis and antibiotic-associated stomatitis [11].

However, under certain conditions, some of the oral bacteria can transform from commensals to opportunistic pathogens when host defense mechanisms fail. Periodontopathogens included in the red complex, Porphyromonas gingivalis, Tannerella forsythia, and Treponema denticola are key periodontopathogens associated with the progression of periodontitis. It is a chronic inflammatory disease that leads to the destruction of the periodontal ligament, alveolar bone resorption, and finally tooth loss [4]. The disease pathogenesis is attributed to these bacteria that evade host immune responses, produce virulence factors (i.e. lipopolysaccharides, gingipains, and collagenases), and induce the release of pro-inflammatory cytokines that exacerbate tissue destruction. C. albicans can cause several simple skin and mucosal infections and potentially fatal deep infections in immunocompromised patients when the fungus switches from commensal to pathogen when there is a shift in the oral environment [13]. Human oral viral interactions span from subclinical colonization to overt clinical diseases. For example, Epstein-Barr virus infection is related to hairy leukoplakia, oral hairy polyps, as well as oral squamous cell carcinoma [10].

In summary, the oral microbiome relies heavily on bacteria, which play crucial roles in maintaining oral health. Disruption in the homeostasis between a commensal microbiota residing in the mouth and the host immune response, owing to various factors, results in dysbiosis that leads to a range of oral diseases. Other populations that actively contribute to oral health and disease include the activities of the oral mycobiome and virome. Unraveling the complexity of polymicrobial nature is essential for the development of preventive and treatment strategies against oral infection.

Microbial Roles in Oral Health and Disease: Mechanisms and Management

The oral cavity is a habitat for a large number of microorganisms, as shown in Table 1, that play an important role in overall health. Streptococcus mutans, a bacterium naturally present in the human oral microbiota; among these, it is one of the most studied opportunistic pathogens involved in dental caries due to its ability to ferment dietary sugars to acids, which leads to dental enamel and dentin demineralization [14]. Excessive growth of S. mutans can be prevented by fluoride treatment and proper oral hygiene. Similarly, Porphyromonas gingivalis, a major periodontal pathogen, causes an inflammatory response that destroys the tissue and periodontitis [15,16]. Deep periodontal cleaning, antimicrobial therapy, as well as host modulation strategies, are effective in managing P. gingivalis overgrowth.

**Table 1:** The microbe type, common species, role in oral health, role in oral disease, mechanism of action, and potential treatment/management Table credit: P. Bhagya Latha

Microbe Type	Common Species	Role in Oral Health	Role in Oral Disease	Mechanism of Action	Potential Treatment/Management
Bacteria	Streptococcus mutans	Part of natural oral flora	Causes dental caries	Produces acid that demineralizes tooth enamel	Fluoride treatment, regular brushing
Bacteria	Porphyromonas gingivalis	Limited in healthy gums	Associated with periodontitis	Promotes inflammation and destroys gum tissue	Deep cleaning, scaling, antibiotics
Bacteria	Lactobacillus species	Contributes to saliva production	May contribute to caries in an acidic environment	Lowers oral pH, aiding in enamel erosion	Probiotics, maintaining neutral pH
Fungi	Candida albicans	Normally controlled by the immune system	Causes oral candidiasis (thrush)	Overgrowth in immunocompromised or antibiotic-treated individuals	Antifungal medication (e.g., nystatin)
Fungi	Candida glabrata	Commensal organism in low levels	Resistant to many antifungals, opportunistic pathogen	Adheres to oral mucosa and biofilm formation	Antifungal treatment, improved oral hygiene
Viruses	Herpes simplex virus (HSV)	Typically latent in nerve tissues	Causes oral herpes (cold sores)	Reactivates under stress or immunosuppression	Antiviral medications (e.g., acyclovir)
Viruses	Human papillomavirus (HPV)	May remain dormant with no symptoms	Linked to oral and oropharyngeal cancers	Infects epithelial cells, causing abnormal growth	HPV vaccination, regular screening
Bacteria	Treponema denticola	Found in small quantities in healthy mouths	Linked to periodontal disease	Produces enzymes that degrade host tissue	Antibiotics, professional cleaning
Fungi	Aspergillus species	Rarely pathogenic in healthy individuals	Can cause oral aspergillosis in immunocompromised patients	Invades oral tissues, forming painful lesions	Antifungal treatment, improved immunity

Besides their bacterial role, Lactobacilli participate in the production of saliva and oral homeostasis, but the overproduction of acid by some strains can decrease the oral pH, thus making individuals susceptible to enamel erosion and tooth decay [17]. As such, probiotic supplementation has been explored as a potential approach to restore pH balance and suppress cariogenic Lactobacilli. In addition, recent research indicates that probiotic and prebiotic interventions have the potential to modulate the oral microbiome lowering pathogenic bacterial loads and therapeutic implications to better oral health outcomes [15].

The oral microbiome is maintained by the immune system to keep fungal homeostasis. Candida albicans is an opportunistic fungal pathogen that is in equilibrium with host immune defenses under normal conditions. In people with compromised immunity, for example, people with HIV/AIDS, or diabetes, who are undergoing chemotherapy, or receiving immunosuppressive treatments, C. albicans overgrowth can cause oral candidiasis (thrush) [18]. Antifungal medications (nystatin) are the standard treatment. Candida glabrata is another opportunistic fungal species that is capable of forming strong biofilm and is increasingly resistant to antifungal drugs in immunocompromised individuals. Management is with proper oral hygiene along with specific antifungal therapy.

Oral pathology is also affected by viral infections. The herpes simplex virus (HSV) is latent in nerve tissues, becomes reactivated with stress or immune suppression, and can result in recurrent lip and oral mucosa cold sores [19]. Outbreaks can be managed using antiviral medications including acyclovir. Besides, human papillomavirus (HPV) has been strongly associated with the development of oral and oropharyngeal cancers such that vaccination programs and early diagnosis screening of at-risk individuals are essential [20].

Low concentrations of other bacterial species, such as Treponema denticola, are present in healthy individuals, but their numbers increase in dysbiotic conditions and they contribute to periodontal destruction [21]. Although limited effect in removing T. denticola can be achieved by conventional treatment of antibiotics and professional dental cleaning, novel therapeutic approaches of antimicrobial peptides and targeted microbiome modulation are required. Aspergillus species are rarely the cause of oral disease in immunocompetent individuals but can cause severe oral infections in patients with, for example, leukemia, organ transplantation, or chronic lung disease [22]. Management requires a combination therapy involving immune support of the immune system and antifungal medications.

Disease susceptibility depends greatly on several host factors, including genetic predisposition, variability in immune response, and lifestyle habits such as smoking, diet, and oral hygiene, among others. The promise of emerging interventional approaches, namely microbiome-based therapies, probiotic formulations, and precision medicine strategies to mitigate the impact of pathogenic oral microbes is presented. These approaches need further clinical research to validate the efficacy of these approaches in long-term prevention and treatment of oral disease.

Oral microbiome and local health impacts

Atherosclerotic Plaques

Recent scientific studies have explored the relationship between certain oral bacteria and atherosclerosis, a condition characterized by the formation of plaques in arterial walls [23]. Several proposed mechanisms explain how oral bacteria may contribute to atherosclerotic cardiovascular disease.

Endodontic bacteria and key periodontal pathogens, including P. gingivalis, A. actinomycetemcomitans, and T. forsythia, have been identified in atherosclerotic lesions [24]. One pathway involves the direct transmission of bacteria to the surface of plaques. These bacteria may enter the bloodstream through open sores in inflamed gums and, using angiogenic factors, penetrate the arterial lining [25]. Once inside the plaques, bacteria trigger inflammation, disrupt plaques, and enhance clot formation [26].

Another mechanism involves immune responses and autoimmunity. Oral bacteria can induce chronic inflammation and promote the formation of autoantibodies, weakening the arterial lining [27]. Additionally, bacteria may provoke cross-reactive antibodies that target host tissues due to antigenic similarity between bacterial and host proteins, a phenomenon known as molecular mimicry [28].

Emerging evidence suggests that oral pathogens can invade arteries, trigger infection and inflammation in atherosclerotic lesions, and contribute to the progression of cardiovascular disease (CVD). Further research is needed to identify optimal treatment strategies for these conditions.

Pneumonia

The human oral cavity harbors a complex community of microorganisms collectively referred to as the oral microbiome. Current research highlights a connection between shifts in the oral microbial balance and diseases affecting distant parts of the body. A notable local health impact is aspiration pneumonia caused by oral microorganisms invading the lungs [29].

Another significant bacterium associated with periodontal infections and detected in the lungs of pneumonia patients is an anaerobic species. These bacteria may temporarily inhabit the oral cavity, invade regions beneath the gingival line, enter the bloodstream, and reach the lungs through aspiration. Additionally, Streptococcus constellatus, a common oral commensal, can become invasive under certain conditions. Strains of S. constellatus isolated from respiratory tract infections exhibit cytopathic effects and invasive characteristics [30].

Individuals with weakened immune systems are at greater risk of pneumonia due to the migration of harmful bacteria from the gums [31]. Maintaining good oral hygiene and practices may help prevent pneumonia, as dysbiosis and the excessive growth of pneumonia-causing oral microbes, which can be inhaled into the lungs, are contributing factors. Further studies are recommended to explore strategies for optimizing the oral microbiome to support overall systemic health [32].

Rheumatoid Arthritis

The oral microbiome refers to the collection of microorganisms inhabiting the oral cavity, including bacteria, viruses, fungi, and protozoa. Recent studies have indicated an association between fluctuations in the oral microbiome and several systemic diseases, including rheumatoid arthritis (RA) [33]. Two bacterial species, Prevotella intermedia and Tannerella forsythia, were found in higher abundance in RA patients compared to control subjects [34].

P. intermedia is a Gram-negative, obligate anaerobe commonly found in periodontal pockets of patients with periodontal disease [35]. Research has shown that the bacterium can spread to the joints from the mouth through bacteremia, triggering an inflammatory response from the immune system [36]. Some studies show that the immune system produces autoantibodies that target joint tissues, leading to swelling and joint deterioration [37]. The incidence of P. intermedia was found to be higher in RA patients compared to the control group (46.7% vs. 26.7%; p = 0.031), suggesting an association between RA and P. intermedia.

In addition, Tannerella forsythia has been linked to susceptibility to RA. This bacterium secretes proteolytic enzymes that likely aid its spread and exhibit immunomodulatory effects [38]. T. forsythia was detected in the synovial fluid of RA patients [39]. Understanding and managing oral dysbiosis can help alleviate symptoms.

*Periodontal Disease* 

The oral microbiome refers to the collection of microorganisms found in the oral cavity and the structures that surround it. These include bacteria, archaea, fungi, protists, and viruses [40]. Periodontal disease manifests due to the effects of various pathogens that normally inhabit the oral cavity. In particular, “red complex” bacteria, including Porphyromonas gingivalis and Tannerella forsythia, have been associated with the disease. These bacteria, specifically T. denticola, form pathogenic biofilms that, following inflammation of the gums (gingivitis), progress to periodontitis. Periodontitis produces pathological pockets in the subgingival area between the gums and teeth, leading to supporting connective tissue and bone loss, which makes the teeth very loose and likely to fall out [41]. An alternative to traditional antimicrobial treatments is antimicrobial photodynamic therapy, which can be used in dysbiotic oral microbiomes [42]. Future studies aimed at understanding how to achieve a healthier oral microbiome could potentially reduce destructive forms of periodontal disease and improve both oral and systemic health.

Primary Endodontic Infection

Pulpal infections, which originate inside the root canals, occur due to dislodging carious processes, trauma, or cracks that expose the pulp tissue [43]. These infecting bacteria are part of the microbial ecosystem of the oral cavity, which includes several hundred species. The microorganisms traditionally causing primary endodontic infections are anaerobic Gram-negative rods such as Porphyromonas spp. and Prevotella spp.; Gram-positive cocci such as Peptostreptococcus; and spirochetes such as Treponema denticola. When the canal becomes infected, acute inflammation of the tissues surrounding the root canal occurs. This can result in severe, spontaneous pain as pressure rises within the inelastic tooth [44]. Since teeth contain nerves, they are a very sensitive part of the body. Therefore, primary endodontic infections can become extremely painful and affect the patient’s quality of life if left untreated [45]. Specific endodontic procedures, such as root canal treatment, are needed to remove the infected pulp, clean the canals, and shape the internal structure of the tooth for healing while keeping the structure intact [46].

Dental Caries

Alterations in the oral microbiota are directly associated with both oral and systemic health. Among the oral diseases caused by microbial imbalance, dental caries, or tooth decay, is one of the major [47]. Dental caries occur when acids generated by cariogenic microorganisms in dental plaque attack the tooth enamel [48]. This results in what is referred to as a cavity. Cariogenic microorganisms include Streptococcus mutans and Lactobacillus spp. [49]. These bacteria ferment dietary carbohydrates and produce lactic acid as a byproduct. This acid dissolves hydroxyapatite crystals in enamel, leading to demineralization and ultimately cavitation. In advanced caries conditions, inflammation and infection can affect the delicate pulp tissue within the tooth. It is clear, however, that S. mutans, Lactobacillus spp., and other cariogenic bacteria play a role in disrupting the natural balance of the oral microbiota, leading to caries development. Ecological concepts and considerations of microbiome interventions are important when designing anti-caries prevention and therapy [50].

Sjögren's Syndrome

Sjögren’s syndrome is an autoimmune disease that manifests as dry eyes and a dry mouth, resulting from damage to the glands that produce tears and saliva [51]. It was postulated that changes in the composition of the oral microbial communities may initiate or worsen Sjögren’s syndrome in genetically predisposed individuals [52]. One potential mechanism is molecular mimicry, in which autoimmune reactions are elicited by oral microbes that are similar in structure or sequence to host tissues, attacking the host’s own moisture-producing glands [53].

Systemic Lupus Erythematosus

The human microbiome consists of a diverse population of microorganisms, including bacteria, fungi, protozoa, and viruses. Oral microbiota are microbes residing in the oral cavity, and they influence the body’s health [54]. Striking changes in the patterns of oral microbial flora have been observed in patients diagnosed with systemic lupus erythematosus (SLE) [55]. SLE is an autoimmune disease that results from the production of autoantibodies and immune complex deposition, affecting the skin, joints, kidneys, CNS, and serosal surfaces [56].

**Figure 2:**
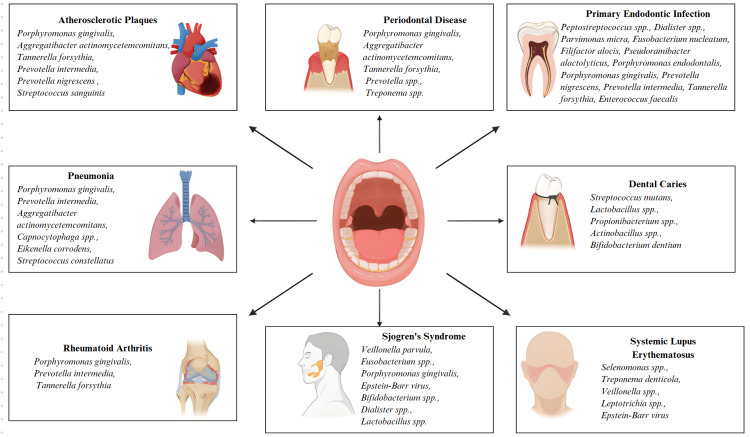
Oral microbiome and its systemic health implications Image credit: Subash Chandra Nayak

Conversion to a new oral microbiota may induce autoimmune reactions through microbial leakage, molecular mimicry, and endotoxin stimulation in genetically predisposed individuals. Specific oral bacteria species found to be related to SLE include Selenomonas spp. and Treponema denticola [57]. Maintaining good oral health may alleviate some symptoms of SLE, as the oral microbiome can influence inflammation and the activation of autoimmune responses [58]. The oral microbiome plays a role in determining SLE risk levels, and its effects on systemic health are depicted in Figure 2.

Oral microbiome and cardiovascular health

Mechanisms

Inflammation, atherosclerosis, and endothelial dysfunction: Gingivitis, periodontal disease, bacterial infections, dental caries, oral dysbiosis, or simply poor oral hygiene are linked to high levels of systemic inflammation and heightened risks of atherosclerosis, coronary artery disease, hypertension, stroke, and cardiovascular inflammation [59]. The pathways by which oral infections might generate cardiovascular risks include increased inflammation, impaired endothelial function, and accelerated atherosclerosis [60]. These bacteria (P. gingivalis and S. mutans) can also penetrate blood vessels and induce the production of cytokines, metalloproteinases, NF-κB, and ROS [61]. This results in endothelial cell activation and injury, low bioavailability of nitric oxide (NO) production, endothelial hyperpermeability, adhesion molecule upregulation, endothelial dysfunction, and impaired repair [62]. It also contributes to the formation of foam cells, plaque development, arterial wall stiffening, and an increase in the thickness of the artery walls over time [63].

Preventive measures include dental checkups and the cleaning of tooth surfaces and root surfaces through scaling and root planning, as well as brushing, flossing, the use of fluoride agents, statins, aspirin, antibiotics, especially probiotics, anti-inflammatory drugs, and antioxidant therapy. Controlling diets to reduce cardiovascular risks is also essential [64]. Caring for gums and other structures in the oral cavity while monitoring oral pathogen loads, addressing them, and managing inflammation through dental and possibly medical approaches can reduce the most prevalent forms of cardiovascular disease risk, as outlined in Table 2.

**Table 2:** Impact of oral health on atherosclerosis and cardiovascular diseases: inflammatory mediators, mechanisms, and preventive approaches IL: interleukin; TNF: tumor necrosis factor Table credit: P. Bhagya Latha

Oral Health Factor	Systemic Condition	Inflammatory Mediators	Mechanism Leading to Atherosclerosis	Endothelial Dysfunction Mechanism	Preventive/Therapeutic Approach
Periodontal disease	Cardiovascular disease (CVD)	C-reactive protein (CRP), IL-6	Chronic inflammation promotes plaque buildup	Reduced nitric oxide (NO) production	Periodontal treatment, statins
Bacterial infection (P. gingivalis)	Atherosclerosis	TNF-α, IL-1β	Bacteria enter the bloodstream, contributing to plaque formation	Increased oxidative stress	Antibiotics, dental hygiene
Dental caries	Systemic inflammation	Prostaglandins, IL-17	Inflammation leads to vascular damage	Endothelial cell damage	Fluoride treatment, oral hygiene
Chronic gingivitis	Coronary artery disease	Matrix metalloproteinases (MMPs)	Degradation of collagen, enabling plaque deposition	Impaired endothelial repair mechanisms	Scaling and root planing, anti-inflammatory drugs
Oral dysbiosis	Hypertension	IL-6, TNF-α	Induces arterial stiffening	Promotes vascular resistance	Probiotics, maintaining oral microbiome balance
Periodontal pathogens	Stroke	Lipopolysaccharides (LPS), IL-8	LPS promotes foam cell formation, leading to thickened arteries	Endothelial permeability, leukocyte adhesion	Periodontal therapy, aspirin
Streptococcus mutans	Cardiovascular inflammation	NF-kB activation	Bacterial invasion promotes arterial inflammation	Endothelial activation, increased adhesion molecule expression	Oral hygiene, anti-inflammatory medications
Poor oral hygiene	Atherosclerosis	Elevated cytokines, IL-1β, TNF-α	Systemic inflammation accelerates atherogenesis	Endothelial apoptosis, vascular rigidity	Regular dental check-ups, diet control
Oral infections	Endothelial dysfunction	Reactive oxygen species (ROS)	ROS damages blood vessel walls	Endothelial nitric oxide synthase (eNOS) inhibition	Antioxidant therapy, improved immunity

Oral microbiome and respiratory diseases

Pathways From the Oral Cavity to the Lungs

**Figure 3:**
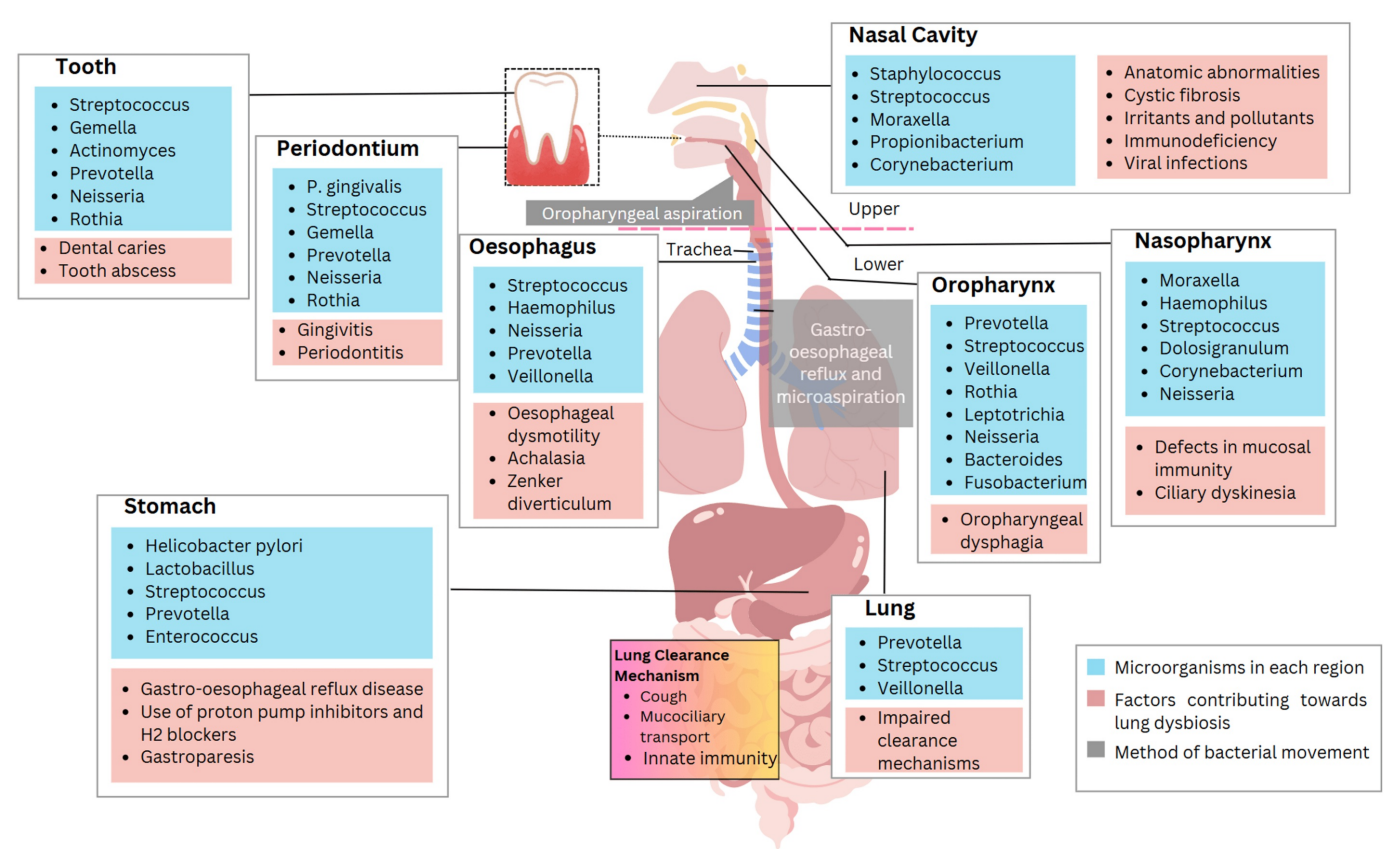
Microbiome distribution and factors contributing to dysbiosis in the human respiratory and digestive tracts Image Credit: Subash Chandra Nayak

Further studies are required to elaborate on how the oral microbiota relates to systemic diseases, as well as to create microbiome-based diagnostic and treatment approaches [65,66]. Several key areas should be prioritized in future studies:

Non-bacterial oral microbes: Although research on oral bacteria has dominated most publications, oral microbiota encompass more than just bacteria. Similar to the mycobiome, virome, archaeome, and parasitome in the oral cavity, these also play a role in systemic health, although the exact nature of this relationship remains undefined [67]. Further research should employ multi-kingdom sequencing and bioinformatics to shed more light on the other microbes that link oral and systemic diseases, as shown in Figure 3. 

Host-microbiome interactions: An in-depth exploration is needed of the immunological and biochemical affiliations between oral microbiota and host tissues, which can potentially trigger systemic inflammation and destabilization of distant solid tissues [68]. Peculiar host-microbe interactions may be elucidated using multi-omics approaches, combining genomics, transcriptomics, proteomics, and metabolomics in clinical cohorts.

Genetics and lifestyle: Genetic predisposition and primary indicators of an individual’s health, such as diet, smoking habits, and alcohol consumption, seem to influence oral microbial ecology and metabolic potential [69]. Future studies should also focus on understanding how genetic and environmental factors shape the specific and unique patterns of oral microorganisms and their association with diseases.

Animal models: Robust animal models that demonstrate the pathophysiology of the association between oral dysbiosis and systemic diseases are still critically needed [70]. These could be developed through microbiome manipulation in xenogenic or immunocompromised mice, such as gnotobiotics or humanized mice, which could trigger enhanced testing of diagnostic biomarkers and treatment approaches affecting the oral microbiome.

Clinical utility: Indeed, relative to other types of diagnostics or interventions based on microbial communities, very few oral-microbiome-based diagnostics or interventions have progressed to the clinic thus far [71]. For future expansion, these studies are required to establish evidence on the effectiveness, the substantial increase in therapeutic value, and the value in the healthcare economy of modulating the oral microbiota in relation to various systemic diseases through appropriately powered randomized controlled trials and cost-utility analyses. 

Interventional studies: While studies have explored the effects of therapies designed to modify the oral microbiome to prevent or treat diseases, clinical investigations remain limited [72]. Randomized Phase II/III trials are now underway to assess the safety and efficacy of orally delivered microbiome biotherapeutics (e.g., prebiotics, probiotics) and should become the highest priority for the field.

Factors Contributing Towards Dysbiosis

Dysbiosis is defined as the disruption of the homeostatic balance of microorganisms in the human body [73]. Various factors affect the balance in each respective area of the human body, which in turn results in the presence of pathogenic bacteria.

Periodontal disease in the oral cavity promotes the overgrowth of bacteria that contribute to gum inflammation and the development of cavities [74]. This indicates that factors affecting the amount of saliva-such as antihistamines or diuretics-also affect the composition of the microbial community in the oral cavity. In the esophagus, gastroesophageal reflux disease (GERD) introduces stomach acid, which favors the flourishing of acid-resistant species. Conditions such as hiatus hernias are predisposing factors for this reflux.

Throughout the gastrointestinal tract, antacid medications decrease stomach acidity, which is essential for preventing microbial growth from the oral cavity, thereby potentially increasing pathogen proliferation and infection risk [75]. Proton pump inhibitors (PPIs) also have a similar dysbiotic effect by suppressing gastric acid secretion. Primary immunodeficiencies, as well as immunosuppression due to medications, hinder the immune system's ability to control pathogen outgrowth and translocation.

Method of Bacterial Movement

There are several routes through which bacteria can migrate from one part of the body to others, primarily due to infections or disruptions in microbial homeostasis [76]. One such process is oropharyngeal aspiration, in which bacteria from the mouth are inadvertently drawn into the lungs [77]. This can occur in healthy individuals but is more frequently observed in the elderly, stroke patients, those who are intoxicated, and patients with dysphagia or an altered gag reflex [78]. Inhaled oral bacteria can lead to aspiration pneumonia or secondary lung infections [79].

Another method is hematogenous spread, the process by which bacteria spread through the bloodstream. Bacteria can enter the bloodstream through direct invasion (e.g., injury), invasive procedures, or translocation across the gut [80]. Once in circulation, bacteria can spread widely and cause infections in other locations such as the heart, joints, meninges, or brain [81]. Some gastrointestinal pathogens, such as Listeria monocytogenes, have a particular affinity for hematogenous spread [82].

Similarly, the urinary tract can become seeded with bacteria originating from the bowel through structural and immunological links between the digestive and urinary systems [83]. Changes in urinary catheters or urological procedures can introduce new bacteria, leading to dysbiosis or clinically evident urinary tract infections.

Lung Clearance Mechanisms

The airways of the lungs have evolved sophisticated strategies to prevent the deposition of particles, allergens, microbes, and toxins inhaled during respiration [84]. The mucociliary escalator facilitates the transport of mucus using ciliated epithelial cells and airway surface liquid, which moves mucus upward from the lungs to the throat, where it can be swallowed to eliminate it from the body [85]. Reduced mucus clearance can lead to infections and lung tissue diseases, such as cystic fibrosis, chronic obstructive pulmonary disease, and bronchiectasis [86].

Coughing effectively eliminates particles and mucus trapped in the lungs. When irritant particles contact cough receptors in the epithelial layer lining the respiratory tract, signals are sent to the brain, triggering a forceful expulsion of the irritants in the form of a cough [87]. Other lung defense mechanisms include physical barriers and the development of innate immunity. Epithelial cells are tightly connected by junctions that restrict the movement of most inhaled materials [88], and macrophages sweep the airways, engulfing pathogens and activating other immune cells when necessary [89]. Maintaining the proper function of these lung clearance systems is essential for optimal respiratory health.

Oral microbiome and pregnancy outcomes

Periodontal Disease and Preterm Birth

The relationship between pregnancy and a woman’s oral health has gained increasing research attention in recent decades. Research has revealed links between specific diseases, including periodontal disease in pregnant women, and adverse outcomes such as preterm birth and low birth weight [90]. As the concept of oral microbial ecology evolves, new parameters linking oral health to pregnancy complications have been discovered.

How Periodontal Disease Risk Contributes to Preterm Birth

Periodontal disease is a chronic and harmful inflammation of the tissues supporting the teeth [91]. The disease originates from microbial imbalances, particularly a dysbiotic oral microbiome, which leads to the deterioration of periodontal tissues [92]. Current literature shows that periodontal disease during pregnancy is associated with preterm birth, defined as birth before 37 weeks of gestation [93]. Proposed mechanisms include hematogenous translocation of microbes and their virulence factors, as well as inflammatory cytokines originating in the oral cavity during early pregnancy, transmitted from mother to fetus via amniotic fluid and the placenta [52]. Such stimulation may provoke immune reactions, leading to premature delivery.

For example, a 2007 prospective cohort study involving over 800 pregnant women assessed pregnancy outcomes and periodontal disease status throughout pregnancy. Women with moderate to severe periodontal disease were 4.5 times more likely to give birth to a very preterm baby (before 32 weeks) compared to women with healthy gums [94]. The severity of periodontal disease was positively correlated with increased risk, showing a dose-response relationship. Subsequently, additional systematic reviews have contributed to the relationship between maternal periodontal disease and higher preterm birth rates [95].

Other intervention trials on treating women’s periodontal disease during pregnancy have shown improvements in some perinatal outcomes. A randomized study conducted in Chile found that integrated periodontal treatment during pregnancy resulted in more than a 50% reduction in preterm birth rates compared to women who received only routine oral care instructions [96]. However, further studies are needed to derive evidence-based data on the most suitable periodontal disease interventions and their effectiveness in achieving optimal pregnancy outcomes [97].

Mechanisms of Oral Infections Leading to Pregnancy Complications

Several adverse effects on both the mother and her unborn child are related to oral infections during pregnancy. Pathogens serving the oral environment, such as P. gingivalis, F. nucleatum, S. mutans, and C. albicans, decline the host inflammatory reactions and enhance cytokines production of IL-6, TNF-α, and CRP in Table 3. These cytokines and mediators are involved with preterm birth, low birth weight, preeclampsia, fetal growth restriction, and neonatal infection.

**Table 3:** Oral Infections, common pathogens, inflammatory mediators, and their impact on pregnancy: mechanisms and preventive/therapeutic approaches IL: interleukin; TNF: tumor necrosis factor Table credit: P. Bhagya Latha

Oral Infection Type	Common Pathogen	Inflammatory Mediators	Impact on Pregnancy	Mechanism of Action	Preventive/Therapeutic Approach
Periodontitis	Porphyromonas gingivalis	IL-6, TNF-α, C-reactive protein (CRP)	Preterm birth, low birth weight	Systemic inflammation, increased prostaglandins	Periodontal treatment, scaling, and root planing
Gingivitis	Fusobacterium nucleatum	IL-1β, TNF-α	Preterm labor, fetal growth restriction	Bacterial translocation to placenta, triggering immune response	Regular oral hygiene, antimicrobial mouthwash
Dental caries	Streptococcus mutans	IL-6, IL-8	Preeclampsia	Increased systemic inflammation and oxidative stress	Fluoride treatment, diet control
Oral dysbiosis	Multiple bacterial species	TNF-α, IL-17	Miscarriage, preterm birth	Microbial imbalance triggers chronic inflammatory response	Probiotics, maintaining microbiome balance
Periodontal abscess	Prevotella intermedia	IL-6, MMPs	Low birth weight, preeclampsia	Local infection spreads to systemic circulation, inducing inflammatory cascade	Antibiotics, drainage of abscess
Oral candidiasis (thrush)	Candida albicans	Th17 cells, IL-23	Premature rupture of membranes	Fungal infection leads to elevated cytokines, weakening fetal membranes	Antifungal treatment, oral hygiene
Herpetic gingivostomatitis	Herpes Simplex Virus (HSV)	IL-1β, IL-18	Neonatal herpes, miscarriage	Viral reactivation and transmission to fetus through placenta or birth canal	Antiviral therapy, suppressive treatment during pregnancy
Periodontal infection	Aggregatibacter actinomycetemcomitans	IL-6, prostaglandins	Preterm labor	Elevates prostaglandin E2 levels, stimulating uterine contractions	Non-steroidal anti-inflammatory drugs (NSAIDs), dental care
Gingival bleeding (inflammation)	Treponema denticola	TNF-α, IL-8	Preeclampsia, placental inflammation	Endotoxins promote systemic inflammation, increasing placental inflammation	Regular dental check-ups, anti-inflammatory medication

Periodontitis is defined as the chronic bacterial infection and inflammation of the tissues surrounding the teeth, prevalent globally. P. gingivalis has been found to induce high systemic concentrations of IL-6, TNF-α, and CRP which facilitate preterm delivery and lower newborn birth weight [98]. Hence, therapies focus on expert prophylactic scaling and root planning, dental profiler, and where possible the use of antimicrobials or host modulators that would help to eliminate bacteria associated with periodontal diseases. Periodontitis initiated by F. nucleatum also increases local cytokines such as IL-1β and TNF-α; predisposing factors to preterm birth and small for gestational age neonates [99]. Intermittent antimicrobial mouthwash and regular professional care of teeth reduce gingival inflammation during pregnancy [100].

Tooth decay, which is initiated by acid-forming Streptococcus mutans, is also a common oral disease [6]. Caries led to higher IL-6 and IL-8 concentrations, amplifying risks of preeclampsia probably through higher systemic inflammation and oxidation. Closing caries needs dietary modification, fluorides, cleanliness, sealants, and prosthetic restorations [101]. Oral thrush is a fungal infection that results from the outcompeting of C. albicans, particularly in immunocompromised persons. Significant amounts of Th17 cells are found in combination with elevated levels of IL-23 and different cytokines which can result in early membrane separation. Fluconazole or clotrimazole with topical antifungal agents, together with mouth-wash for thrush has been advised [102].

In general, various oral pathogens might be deleterious to both maternal and fetal health depending on the extent of local and/or systemic inflammation. Periodontal disease and other oral infections should be treated; active infections should be treated and infection risk should be decreased; oral hygiene should be explained and reinforced; diet and microbiome balance should be managed to prevent complications of periodontal diseases during pregnancy [103].

## Conclusions

Our findings highlight the importance of the oral microbiome in human health and the tight junction between dentistry and medicine. Although cardiovascular diseases remain a significant area of research, there is increasing evidence supporting the potential role of oral microbiota in disease processes such as diabetes, respiratory diseases, and pregnancy as well as neurodegenerative disorders. Thus, oral health is an important part of general health and must be recognized as a whole, rather than as a problem apart. Microbiome-targeted interventions (i.e., probiotic therapies, antimicrobial peptides, precision medicine) offer means to enhance patient outcomes in breast cancer. However, additional clinical research is necessary to define causal relationships between oral microbiota dysbiosis and particular systemic conditions. To validate such microbiome-based therapeutic strategies, large-scale longitudinal studies and randomized controlled trials are needed.

From a clinical standpoint, bringing together oral healthcare within routine medical practice, forming interprofessional collaborations between dentists, physicians, and microbiologists, and carrying out preventive oral health measures in at-risk populations could decrease the chronic disease burden by far. Therefore, oral health assessments must be a standard practice of systemic disease prevention and management in future healthcare models. Filling the gap between dentistry and general medicine allows greater practicability of more effective, evidence-based interventions to improve both oral and general well-being.
